# Images of God and attitudes towards death in relation to spiritual wellbeing: an exploratory side study of the EORTC QLQ-SWB32 validation study in palliative cancer patients

**DOI:** 10.1186/s12904-017-0251-7

**Published:** 2017-12-08

**Authors:** Renske Kruizinga, Michael Scherer-Rath, Johannes B. A. M. Schilderman, Mariëtte Weterman, Teresa Young, Hanneke W. M. van Laarhoven

**Affiliations:** 1 Department of Medical Oncology, Academic Medical Center, University of Amsterdam, Meibergdreef 9, F4-261, 1105 AZ Amsterdam, The Netherlands; 2 Faculty of Philosophy, Theology and Religious Studies, Radboud University Nijmegen, Erasmusplein 1, 6500 HD Nijmegen, The Netherlands; 3 Lynda Jackson Macmillan Centre, Mount Vernon Cancer Centre, Northwood, Middlesex, UK

**Keywords:** Spirituality, Wellbeing, Spiritual wellbeing, Oncology, Images of God, Attitudes towards death, Palliative care

## Abstract

**Background:**

When patients are facing the ends of their lives, spiritual concerns often become more important. It is argued that effective, integrated palliative care should include addressing patients’ spiritual wellbeing. In 2002 the EORTC Quality of Life Group began an international study to develop an spiritual wellbeing measure for palliative patients (SWB). Spiritual wellbeing is a complex construct, which comprises multiple contributory components. While conducting the EORTC SWB validation study with Dutch palliative cancer patients we also conducted an exploratory side study to examine the relationship between their spiritual wellbeing, images of God, and attitudes towards death.

**Methods:**

Patients with incurable cancer who were able to understand Dutch and were well enough to participate, completed the provisional SWB measure and two scales assessing “Images of God” and “attitudes towards death and afterlife”. Linear stepwise regression analysis was conducted to assess the relation between SWB and other factors.

**Results:**

Fifty two Dutch patients, 28 females and 24 males, participated. The whole SWB measure validation identified four scoring scales: Existential (EX), Relationship with Self (RS), Relationships with Others (RO), Relationship with Something Greater (RSG) and Relationship with God (RG, for believers only). Adherence to an image of an Unknowable God and a worse WHO performance status were negatively associated with the EX scale. The image of an Unknowable God was also found to be negatively associated with the RS scale. Higher education correlated positively with the RO scale. Adherence to a Personal or Non-Personal Image of God was not found to be positively influencing any of the domains of SWB.

**Conclusions:**

For our participants, an Unknowable Image of God had a negative relationship with their SWB. Furthermore, specific images of God (Personal or Non Personal) are not associated with domains of SWB. Together, these findings suggest that spiritual wellbeing surpasses traditional religious views. The development of a new language which more naturally expresses different images of a higher being amongst patients in western late-modern societies may further aid our understanding and subsequently lead to an improvement in patients’ spiritual wellbeing.

## Background

When patients face the ends of their lives, spirituality may become more important [1]. Questions like what ‘what is the meaning of my suffering?’ and ‘is there life after death?’ become more pressing when people know they do not have much time left [2]. Cicely Saunders, the founder of the modern hospice movement, argued that dying people experienced “total pain” - physical, social, emotional and spiritual; and the World Health Organization states that palliative care should integrate psychological and spiritual aspects of patient care and aim at enhancing quality of life [3–5]. Thus, assessing patients’ spiritual wellbeing, including trying to understand the concept of spirituality, is crucial for effective, integrated palliative care [6, 7]. In 2009 a Consensus Conference with the aim to improve the quality of spiritual care agreed on the following definition: ‘Spirituality is the aspect of humanity that refers to the way individuals seek and express meaning and purpose and the way they experience their connectedness to the moment, to self, to others, to nature, and to the significant or sacred’ [8].

Different measures of spiritual wellbeing have been developed over recent years [9–11], however none of these measures have been developed cross culturally from the outset. In 2002, the European Organisation for Research and Treatment of Cancer (EORTC) Quality of Life Group began an international study to develop a measure of SWB for palliative patients. Pilot-testing was conducted in 6 European countries and Japan [12], and validation field-testing was completed in 2014, with the EORTC QLQ-SWB32 now validated in 14 countries, including the Netherlands [12].

Previous studies have shown that images of God and attitudes towards death are directly or indirectly associated with quality of life, depression and hopelessness in advanced cancer patients in the Netherlands [13, 14]; in addition there is a relationship between specific concepts of religiousness and spiritual wellbeing [15–17]. In the Netherlands, whilst participating in the validation study, we aimed to explore the relationship between spiritual wellbeing as measured by the EORTC QLQ-SWB32, images of God, and attitudes towards death and afterlife.

## Methods

### Inclusion criteria and data collection

From March 2012 to August 2013, patients in a Dutch hospital who had incurable cancer (breast and prostate cancer patients Stage 4, all other solid tumors at least stage 3), were able to understand Dutch, and had a WHO performance score of 0–2 were invited to take part in the Phase IV field-testing and validation of the Dutch version of the provisional SWB measure. Sociodemographic and clinical data were collected for all participants. Participants completed the provisional SWB measure and also two questionnaires on images of God and attitudes towards death and afterlife [13, 14, 18]. The study was approved by the institutional medical ethics board of the Academic Medical Centre Amsterdam.

### Patient recruitment

Recruitment was carried out at the outpatient clinic. Oncologists asked patients if they were willing to participate in the study. After the patients gave their verbal and written consent, the researcher made an appointment with each participant to complete the tools and obtain their oral comments.

### Tools

#### SWB measure

The overall validation study for the EORTC QLQ-SWB32 identified 22 items which contributed to four scoring scales applicable to all respondents: Existential (EX) (e.g. “I feel at peace with myself”); Relationship with Self (RS) (e.g. “I feel lonely”); Relationship with Others (RO) (e.g. “I feel loved by those important to me”); and Relationship with Something Greater (RSG) (e.g. “I believe in life after death”). A further 10 items have clinical utility including identifying those respondents who believe in God or someone/something greater than themselves and for whom a single item scale Relationship with God (RG) is valid. A four-point scale (Not at all - A little - Quite a bit - Very much) is used for all the items, except the final item, which asks respondents to score their overall SWB on a seven-point response scale (from 1 “very poor” to 7 “excellent”) plus the additional option of “0” for do not know or cannot answer.

#### “Images of God”

To assess participants’ images of God we used a 14-item Dutch instrument which has been used in a large survey (*n* = 1008) on Socio-Cultural developments in the Netherlands [19, 20]. We used the factor-structure reported by Van Laarhoven [14] as our sample was similar in terms of patient characteristics, to distinguish three different images of God: a Personal God (God knows and understands me)*;* a Non-personal God (There is something that unifies man and world in their very roots) and an Unknowable God (God (someone/something) surpasses our powers of imagination) [19, 20]*.* The items were scored on a scale from 1 totally agree to 5 strongly disagree and the Crohnbach’s α is .98 for the scale “Personal God”, .93 for “Non-Personal God” and .81 for “Unknowable God” [14].

#### Attitudes towards death and dying

Patients also completed a 27-item Dutch instrument developed by Scherer-Rath [21] on different attitudes towards death and dying. Using the empirical model of van Laarhoven [13] we distinguish five different attitudes: Explicitly religious (God decides about life and death, Crohnbach’s α = .96), Agnostic/Atheistic (not knowing or not believing that there is life after death, Crohnbach’s α = .73), Reincarnation (rebirth of the soul in another form, Crohnbach’s α = .83), Community (reencounter with deceased after death, Crohnbach’s α = .95) and Continuation (I believe in life after death, Crohnbach’s α = .92)*.* Please note, we do acknowledge that agnostic and atheistic views on death and afterlife are fundamentally different. However, in the context of this particular study, we treat the two views as a single concept in their accordance of not explicitly believing in life after death.

All items used a five point scale from totally agree to strongly disagree. For the analysis of the tools ‘Images of God’ and ‘Attitudes towards death and afterlife’ we recoded the items in: 1 strongly disagree to 5 totally agree, so that it was more easily comparable to the SWB scales which range from 1, not at all to 4, very much. The scales within each of these two tools are not mutually exclusive, participants can adhere to multiple images or attitudes even if they are theoretically mutually exclusive.

### Statistics

Patient’s sociodemographic characteristics were also considered as factors that could influence SWB. Associations between images of God, attitudes towards death and the Relationship with Others, Relationship with Self and Existential scales from the SWB measure and socio-demographic factors were first analyzed by Pearson’s correlation analysis. Only the significant associations with *p* < 0.05 were taken up in a stepwise linear regression model. All statistical analyses were performed with SPSS (version 20.0). Statistical inferences were based on 2-sided tests with *p* < .05 considered to be statistically significant. We did not include the Relationship with God scale from the EORTC SWB32 because too few participants completed that scale (*n* = 29) or the scale Relationship to Something Greater because it had too much overlap with the tools regarding images of God and attitudes towards death. Missing data were excluded list wise.

## Results

Fifty two patients, 28 females and 24 males participated in the study. The mean age was 61 years (SD 9.8, Table 1). The majority had no religious affiliation and had a WHO performance status of 1 (able to carry out all normal activity without restrictions (WHO-0), Restricted in physically strenuous activity but ambulatory and able to carry out light work (WHO-1), or Ambulatory and capable of all self-care but unable to carry out any work; up and more than 50% of waking hours (WHO-2)). A range of tumour sites were represented in the sample. Of the three different images of God, most patients adhered to an Unknowable Image of God (God/Someone or something higher surpasses our powers of imagination), even though there is rather strong dispersion regarding the item ‘personal God’. Of the five different attitudes towards death and dying most patients adhered to an agnostic/atheistic attitude (not knowing or believing in life after death). The highest median score across the four scales of the EORTC QLQ-SWB32 was for the RO (‘Relationship with Others’) scale (Table 2). Twenty nine participants identified themselves as somewhat believing in God or someone/something greater than themselves, so they completed the RG scale, 19 participants did not completed the RG scale.

**Table 1:** Patients demographic and disease characteristics

		Number	Percent
Sociodemographic characteristics	Age	61 (mean) 60,3 (median)	9.8 (SD) 38 (range)
	Male	24	46.2
	Female	28	53.8
	Education ≤ compulsory	27	51.9
	Education > compulsory	24	46.2
	Birth country the Netherlands	42	80.8
	Birth country other	10	19.2
	Not working	38	73.1
	Working	13	25
WHO performance status	0: Fully active	12	23.1
	1: Restricted	32	61.5
	2: Ambulatory	7	13.5
Religiosity	Religious	17	32.7
	Not religious	35	67.3
Cancer type	Breast cancer	8	15,4
	Colorectal cancer	11	21,2
	Cholangiocarcinoma	6	11,5
	Pancreatic cancer	9	17,3
	Other solid tumor	15	28,8

**Table 2:** Mean level of adherence to images of God, attitudes towards death and SWB

	Different scales	Mean	SD	Score ≥ 3,5 (total)
Images of God (5 point scale)	Personal God	2,75	1,42	18 (50)
	Non-personal God	2,70	0,75	24 (49)
	Unknowable God	3,23	1,18	19 (46)
Attitudes towards death (5 point scale)	Explicitly religious	2,53	1,34	
	Agnostic/Atheistic	3,35	0,96	
	Reincarnation	2,17	1,08	
	Community	2,43	1,24	
	Continuation	2,70	1,27	
Spiritual wellbeing scales (4 point scale)	Existential wellbeing	2,96	,59	
	Relation to Self	3,05	,54	
	Relation to Others	3,30	,50	
	Relation to Something Greater	2,40	,73	
	Relation to God (1-item scale for ‘believers’ *n* = 29 completed this scale)	2,38	1,02	

To investigate relations between the images of God, attitudes towards death, patient characteristics and the three SWB scales we performed correlation analyses (Table 3). A Personal as well as a Non-personal image of God was significantly correlated with Relationship with Something Greater (0.526 and .438). In contrast, an Unknowable image of God was negatively correlated with Existential Wellbeing and Relation to Self (−0.391 and −0.579). Secondly, all of the attitudes towards death and afterlife scales were significantly correlated with Relationship with Something Greater and -except for the Reincarnation scale- also with Relation to God. These correlations were all highly significant and positive, between 0.442 and 0.678; with exception of Agnostic/Atheistic scale which was −0.399. Amongst the patient characteristics being religious or spiritually involved showed a highly significant correlation with Relationship with Something Greater (0.468). Living with a partner, on the other hand, showed a highly negative correlation to the same scale (−0.370).

**Table 3:** Correlation between SWB and images of God, attitudes towards death and patient characteristics

			Existential (EX)	Relationship with Self (RS)	Relationships with Others (RO)	Relationship with Something Greater (RSG)	Relationship with God (RG)
Images of God	Personal God	r	-,176	-,178	-,209	,526**	,362
		Sig.	,222	,217	,146	,000	,053
	Non-personal God	r	-,243	-,119	-,072	,438**	,073
		Sig.	,092	,188	,624	,002	,713
	Unknowable God	r	-,391**	-,579**	-,254	,015	-,096
		Sig.	,007	,007	,089	,919	,635
Attitudes towards death and afterlife	Explicitly religious	r	-,126	-,139	-,144	,562**	,498**
		Sig.	,382	,335	,319	,000	,006
	Agnostic/Atheistic	r	,002	,000	,215	-,399**	-,370*
		Sig.	,987	,999	,133	,004	,048
	Reincarnation	r	-,215	-,140	-,272	,442**	,161
		Sig.	,133	,332	,056	,001	,403
	Community	r	-,082	-,079	-,153	,580**	,386*
		Sig.	,571	,583	,288	,000	,038
	Continuation	r	,004	-,064	-,110	,678**	,477**
		Sig.	,980	,658	,448	,000	,009
Patient characteristics	Living with a partner	r	-,041	-,238	-,190	-,370**	-,020
		Sig.	,781	,100	,191	,009	,918
	Religious/involved in a spiritual organization	r	,125	,073	,021	,468**	-,229
		Sig.	,388	,612	,883	,001	,232
	Education	r	,240	,234	,322*	,000	-,166
		Sig.	,096	,106	,024	,999	,390
	WHO performance status	r	-,395**	-,326*	-,168	,187	,048
		Sig.	,005	,022	,247	,199	,808

*indicates *p* < .05

** indicates *p* < .01 Pearson’s correlation coefficient r is given; RG is only for believers *n* = 29

For the scales EX, RS and RO we conducted a linear stepwise regression analysis (Table 4), only significant correlations were taken up in the regression model. We found that the image of an Unknowable God and a worse WHO performance status were significant negative predictors for the scale Existential Wellbeing (*p* = .022, b = −,330 and *p* = 0.41, −.291). Adherence to the image of an Unknowable God was also found to be a negative predictor for the scale Relationship with Self (*p* = 0.00 b = −,578). For the scale Relationship with Others, Education was found to be a positive predictor (*p* = 0.024 b = .322).

**Table 4:** Stepwise regression analysis of images of God and patient characteristics with SWB scales

Dependent variable (SWB questionnaire)	model	Independent variables (Images of God, attitudes towards death, patient characteristics)	Standardized coefficients Beta	Sig.	R	R^2^	Adjusted R^2^
Existential	1	Unknowable God	-,391	,008	,391	,153	,133
	2	Unknowable God	-,330	,022	,484	,234	,197
		WHO score	-,291	,041			
Relation to Self	1	Unknowable God	-,578	,000	,578	,334	,319
Relation to Others	1	Education	,322	,024	,322	,104	,084

Stepwise linear regression was performed on only three SWB scales: EX, RS and RO, see methods. Per SWB scale all significant models are shown with the independent variables which were entered successively

## Discussion

This study is the first to show that an Unknowable Image of God might have a negative impact on spiritual wellbeing, as measured by the EORTC SWB32. An Unknowable Image of God entails that one believes that there is a God or Higher Being but one cannot know or cannot relate directly to Him/It. Having an image of a distant God results in not being engaged in a helpful relationship with God, and, therefore, finding meaning and peace in life and finding comfort and strength in difficult situations can be more complicated [22].

One could expect that a Personal Image of God - which implies a more direct relationship with God and therefore more direct sources for finding meaning and peace - would have a positive influence on SWB. In contrast, people who are not engaged in a helpful relationship with God, might have more difficulties by finding meaning and peace in life and finding comfort and strength in difficult situations. Based on our study, this expectation has to be nuanced: a personal Image of God was weakly related to Existential wellbeing, Relationship with Self and Relationships with Others, while it was strongly related to Relationship with Something Greater (and moderately also with Relationship with God). Thus, this implies that a Personal Image of God has a very specific influence on SWB. However, we did not find a positive relation between a Personal or even a Non-Personal Image of God and the scales of SWB as measured with the SWB32. This finding is in contrast with many studies conducted in the USA that all showed positive relations between belief in a Personal God and different forms of spiritual wellbeing [15, 23–25]. Unfortunately, the vast majority of coping and spirituality studies only include personal images of God and do not report on images of a distant or unknowable God. Since the significance of God for daily life has declined in the secularized society of the Netherlands, it may be hypothesized that not many Dutch people adhere to a vividly helpful relationship with a personal God [26, 27]. The finding that most of the participants adhere to an agnostic attitude towards death and afterlife underlines the declined influence of religious salience in our Dutch study population [28, 29].

However, the high scores on the EX, RS and RO scales (Table 2) which were not related to specific images of God or attitudes towards death and afterlife, may indicate that these forms of spiritual wellbeing surpass the traditional religious views. Other studies also support this line of reasoning and identified a growing population who define themselves as ‘spiritual but not religious’ [30–32]. This group may have an aversion to traditional religious concepts, and so avoid the word ‘God’ [30].

Nevertheless, there are indications that experiences of the ultimate or of transcendence may be important factors influencing spiritual wellbeing [33, 34]. Utsch, for instance, states that images of God can be either healing or sickening and are interacting with one’s self-image [35]. Dezutter et al. found that a positive interpretation of one’s disease combined with a positive God image also influences patients’ happiness [36] and Büssing showed in his validation paper [37] that the concepts of ‘search’ and ‘reflection’ have spiritual connotations as they are associated with positive interpretations of illness. Negative feelings towards a higher being/God such as anger, are also associated with less wellbeing [38]. Spirituality thus can have an impact on how patients deal with their life concerns. These experiences though, cannot be captured by the classic interpretation of images of God. Therefore, we have to explore other ways that people use to express their spiritual experiences and their connectedness to ‘a Higher Being’.

Emmons, for instance, found that goal-directed behavior can provide meaning and therefore increases a sense of spiritual wellbeing [39]. By searching for things that best provide a sense of meaning and purpose, he is able to describe the concept of spirituality in goals. Those personal goals are concerned with ultimate purpose, ethics, commitment to a higher power, and a seeking of the divine in daily experience. For instance, ‘to approach life with mystery and awe’ can be a spiritual striving which reflects the desire to transcendent the self, but cannot be captured in a traditional understanding of religiosity [40].

Therefore, a new language or imagery needs to be developed that can express the diversity of spiritual experiences in our western societies and is sensitive to spiritual ideas and experiences independent from traditional religion.

### Strengths and limitations of the study

The identification of factors that influence spiritual wellbeing is an important step towards the development of interventions to improve spiritual wellbeing. This study provides explorative insights into the complex concept of spiritual wellbeing and provides a rationale to develop a new language to indicate the relation with ‘something higher’. The study was conducted in the outpatient clinic of one academic hospital which limits the generalization of the results. Our study population - with a mean WHO score of 1- does not correspond with the palliative patient population in general. The questionnaires were filled in in the presence of the researcher, this might have influenced the data. However, the researcher took great care to stimulate patients to come up with own answers to the questions posed, instead of providing an answer for them. The provisional character of the SWB questionnaire might have been of influence since four questions are no longer part of the final version. For our analysis, however, we also deleted those questions and we analyzed the data as required in the protocol of the final model. Also, as our sample size was relatively small, our results should ideally be validated in a second independent set of data to allow for definite conclusions. It would also be of great interest to compare the results of this study with other patient categories or even healthy persons. In a previous study, we observed that former cancer patients (without evidence of disease) and advanced cancer patients did not differ in attitudes or emotions towards death, but the relation of these attitudes and emotions with aspects of quality of life varied [13].

## Conclusion

An Unknowable Image of God was found to be negatively influencing SWB. Furthermore, specific images of God (Personal or Non Personal) were not found to have a positive influence on SWB. These findings suggest that domains of SWB surpass traditional religious views. The development of a new language and imagery which more naturally expresses different experiences of the transcendent of modern western patients may further aid in understanding and therefore might lead to an improvement in patients’ spiritual wellbeing.

## Abbreviations

EORTC: European organisation for research and treatment of cancer
EX: Existential
RG: Relationship with God
RO: Relationships with others
RS: Relationship with self
RSG: Relationship with something greater
SWB: Spiritual well being
WHO: World Health Organization
